# Epidermoid Cysts in a Wandering Spleen: An Unusual Enigma

**DOI:** 10.1155/2019/1581736

**Published:** 2019-11-20

**Authors:** Sarah E. Algino, Siena Sorrentino, David T. Luyimbazi, Douglas J. Grider

**Affiliations:** ^1^Edward Via College of Osteopathic Medicine, Blacksburg, Virginia, USA; ^2^Virginia Tech, Blacksburg, Virginia, USA; ^3^Department of Surgery, Carilion Clinic and Virginia Tech Carilion School of Medicine, Roanoke, Virginia, USA; ^4^Department of Basic Science Education, Virginia Tech Carilion School of Medicine, Roanoke, Virginia, USA

## Abstract

Epidermoid splenic cysts are rare lesions in the spleen. These cysts are characterized by a stratified squamous epithelial lining, internal septations, and calcification. Congenital in origin, epidermoid splenic cysts are postulated to arise from misfolding and mesothelial cell incorporation into the splenic parenchyma. This report presents a unique case of an 18-year-old woman with an epidermoid splenic cyst in a congenital wandering spleen. Computed tomography and transabdominal ultrasound imaging along with immunochemistry staining confirmed the diagnosis. To the authors' knowledge, this is the first reported case of an epidermoid cyst in a wandering spleen.

## 1. Introduction

Splenic epidermoid cysts are rare, only accounting for around 10% of splenic cysts, and can be described as primary, nonparasitic cysts that are typically congenital in origin [1–3]. Epidermoid splenic cysts are characterized by having an epithelial cell lining composed of stratified squamous epithelium, variable cuboidal mesothelial cells, and rarely mucous cells. This epithelial lining distinguishes primary from secondary cysts or pseudocysts which lack an epithelial lining [3].

Epidermoid splenic cysts most commonly affect individuals of a young age and have varied clinical presentations. Most cases are asymptomatic; however, cysts larger in size may present with abdominal symptoms, such as discomfort, fullness, nausea, vomiting, pain, or a tender palpable mass [1–4]. Abdominal ultrasonography (US) or computerized tomography (CT) are the primary imaging modalities employed for diagnosing splenic epidermoid cysts. However, histological examination is required to make the diagnosis. Thus, partial or total splenectomy is the optimal treatment [3, 4].

An epidermoid splenic cyst is an unencapsulated, usually circumscribed cystic mass located in the spleen. Due to the size of the cyst and its location in splenic parenchyma, splenomegaly is commonly seen on US and CT studies. Grossly, epidermoid splenic cysts are greyish white in appearance. Histologically, a stratified squamous epithelial lining is present lining the cyst wall interior. Internal septations and calcification are variably present. Positive staining for CK5/6 and CEA by immunohistochemistry helps make the diagnosis. If there is a mesothelial flat to cuboidal cell lining component, it will also be CK5/6 positive, along with calretinin or other mesothelial markers by immunohistochemistry [3–5].

Reported is a unique case of an epidermoid splenic cyst discovered in an 18-year-old woman with travel history to Australia and Sardinia, regions endemic to cystic echinococcosis, also commonly known as Hydatid disease.

## 2. Case Report

**Figure 1:**
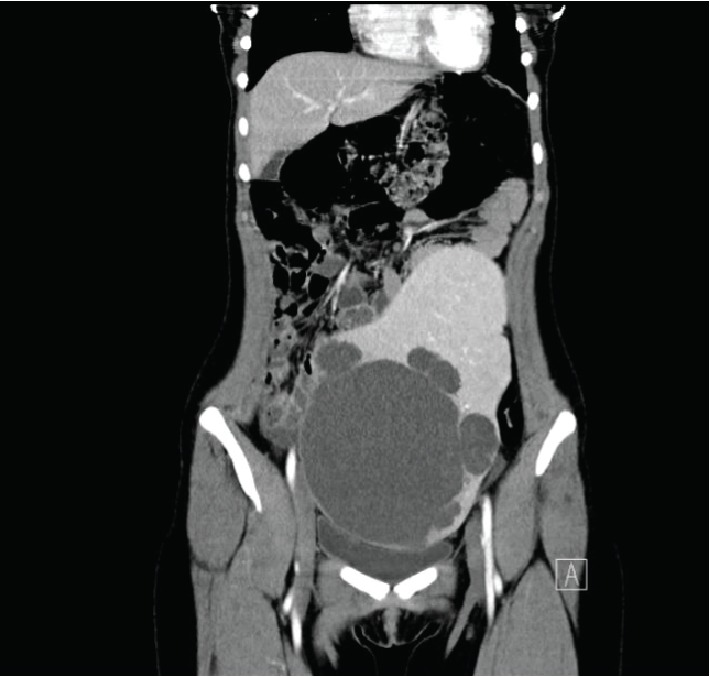
Coronal plane CT image of large intra-abdominal wandering spleen with cystic masses.

An 18-year-old woman with recent travel history to Australia and Sardinia presented with constant lower abdominal fullness for one-year duration, with increased intensity over the last 3-4 months. The patient denied any occurrence of nausea, vomiting, or pain. Transvaginal ultrasound revealed a large cystic mass spanning the abdominal and pelvic regions. The only notable laboratory finding was a mild elevation in tumor marker CA 19-9. Subsequent CT imaging exhibited an enlarged, wandering spleen measuring 24.5 × 16.0 × 12.0 cm, in the patient's left lower abdomen and extending into the anterior mid pelvis (Figure 1). No evidence of venous or arterial obstruction was noted. A 16.0 × 15.7 × 1.4 cm mass in the inferior aspect of the spleen was seen composed of a large central cystic space with several peripheral cystic structures of varying sizes. Minimal calcification was demonstrated along the septations within the lesion. In addition, an 1.7 × 1.5 × 1.6 cmsolid-appearing region was detected at the left mid anterior aspect of the spleen, possibly representing a portion of the vascular pedicle. The patient presented no other notable medical history or physical findings. An open splenectomy was deemed appropriate for treatment in order to prevent torsion of the splenic pedicle.

**Figure 2:**
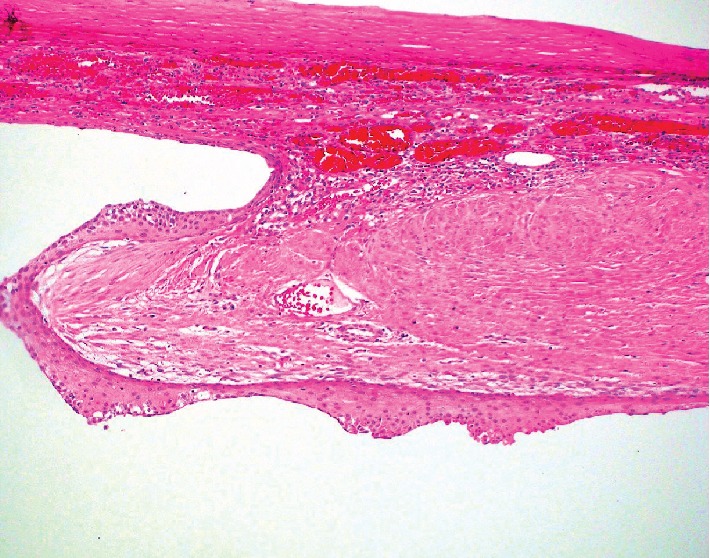
Stratified squamous epithelial lined splenic cyst (epidermoid cyst) (H&E 100 magnification; 10x).

**Figure 3:**
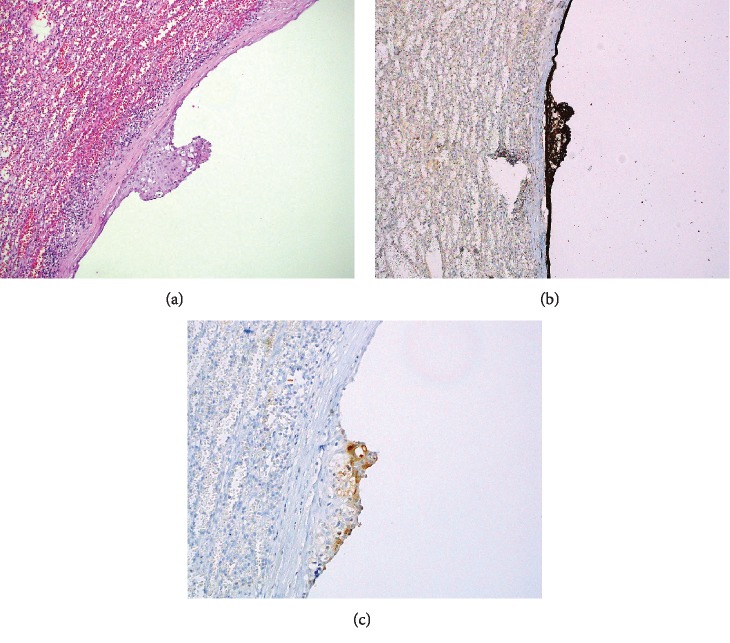
(a) Splenic epidermoid cyst with stratified squamous epithelial with focal mucous cells and simple flat mesothelial cell cyst lining (H&E 100 magnification; 10x). (b) CK5/6 IHC marking both stratified squamous epithelial and simple flat mesothelial cell lining cells (100 magnification; 10x). (c) CEA IHC marking stratified squamous epithelial cells (200 magnification; 20x).

**Figure 4:**
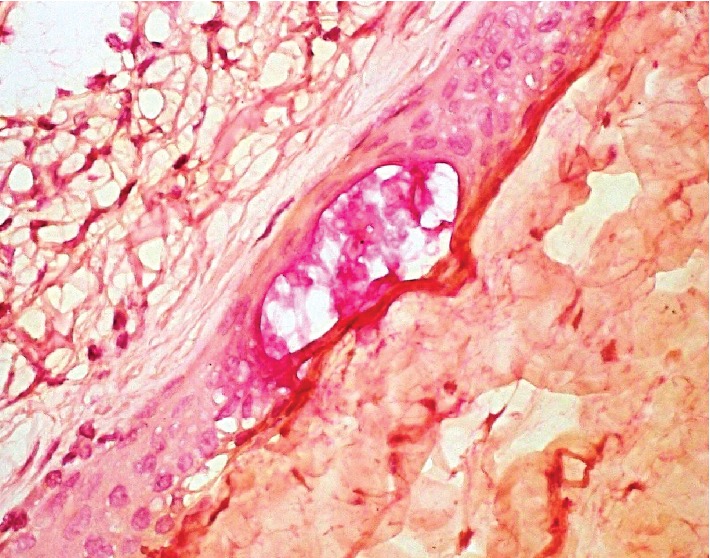
Mucicarmine marking mucin collection in the midst of stratified squamous epithelium in a splenic epidermoid cyst (600 magnification; 60x).

Extirpation of the spleen revealed a circumscribed 16 cm gray-white, smooth, and fibrotic mass. Serial sectioning of the mass showed a large cyst filled with yellow fluid. The lining was smooth, tan and focally hemorrhagic. Many smaller peripheral cystic lesions of variable sizes emanated from this larger cystic space, measuring between 0.4 and 0.3 cm. No echinococcal cysts or papillary excrescences were detected. Microcalcifications were noted in many sites of the cyst wall septations. Histology showed a variable stratified squamous cell epithelium and simple cuboidal to flat mesothelial cell lining on densely fibrotic walls of variably sized cystic spaces. (Figure 2) Immunohistochemistry was positive for CK5/6 and CEA in the squamous lining of the cystic spaces, confirming a splenic epidermoid cyst. (Figures 3(a)–3(c)) The mesothelial cells were also CK5/6 but were also calretinin positive. Mucicarmine focally marked mucous cells within the squamous epithelial lining. (Figure 4) No skin adnexa or other mesodermal or endodermal structures were seen, helping to exclude a dermoid cyst or cystic teratoma. Echinococcus IgG study was negative, helping to exclude a hydatid cyst. Rare areas of stratified squamous epithelium had focal mucous-type cells, positive on mucicarmine-stained tissue sections, a feature also described in epidermoid cysts [6]. The pedicle of the wandering spleen had lymphatic, arterial and venous vessels of variable sizes and somewhat disordered distribution, mimicking a vascular malformation.

## 3. Discussion

When examined, epidermoid splenic cysts appear smooth and grey-white in color grossly. Histologically, they have a minimum of stratified squamous epithelial cyst lining but can also have cuboidal to flat mesothelial cells and rarely mucous cells. Septations or trabeculations within the cyst may also be present. The cysts contain fluid that varies from serous to purulent. Epidermoid splenic cysts are congenital and are postulated to arise during the development of the spleen in utero, specifically from the incorporation of mesothelial cells into the splenic parenchyma [1, 2, 6].

The differential diagnosis includes a variety of benign splenic abnormalities including hydatid cysts, lymphangiomas, and pseudocysts. Hydatid cysts chiefly inhabit the liver and lungs; less commonly, they may also occur in the spleen [7]. Splenic hydatid cysts mirror some of the clinical features of epidermoid splenic cysts, namely, splenomegaly and the presence of a large, circumscribed cystic lesion on abdominal US. However, hydatid cysts present with different clinical history and display cyst wall calcification and hyperdense internal membranes. These cysts are caused by the tapeworm *Echinococcosis granulosis* and are endemic in areas where sheep are raised, such as Asia, Central and South America, Europe, and Africa [7–9]. In addition, clinical presentation of hydatid splenic cysts involve gastrointestinal disturbances such as dyspepsia, gastritis, and constipation. Furthermore, hydatid splenic cysts are hyperechoic on abdominal ultrasound while epidermoid splenic cysts are anechoic [1, 7–9]. Lastly, no stratified squamous epithelial lining is present to suggest epidermoid splenic cysts.

Splenic cystic lymphangiomas, too, present with similar clinical features of splenic epidermoid cysts, most notably splenomegaly, abdominal fullness, and cystic masses appearing on ultrasound imaging. Patients with cystic lymphangiomas are predominately asymptomatic. However, lymphangiomas are unique in that they are filled with eosinophilic, proteinaceous lymphatic fluid [10]. Splenic epidermoid cysts do not contain lymph material, rather they contain serous fluid of varied color. Additionally, histological examination reveals flattened endothelial lining in cystic lymphangiomas, while epidermoid cysts display stratified squamous epithelial lining. Another remarkable factor differentiating splenic cystic lymphangiomas from splenic epidermoid cysts is that lymphangiomas present with dilation of the lymphatic vessels of the splenic parenchyma while no venous abnormalities are typically associated with splenic epidermoid cysts [11, 12].

Splenic pseudocysts, much like the other diagnostic considerations, display commonalities with epidermoid splenic cysts, including similar presentation on imaging; thus, histology is vital in diagnosing pseudocysts. Pseudocysts are sometimes categorized as secondary cysts due to their lack of epithelial lining internally. Epidermoid cysts of the spleen are primarily due to epithelial lining. Histology further shows that pseudocyst walls are lined solely by fibrous tissue, often with associated hemosiderin-laden macrophages [13, 14].

Dermoid cysts of the spleen also known as cystic teratomas should, too, be ruled out when considering cysts of the spleen. Dermoid cysts of the spleen are rare in occurrence and have a similar clinical presentation to previously discussed splenic cysts, consisting of lower abdominal pain, fullness of the abdomen, and a cystic lesion of the spleen present on imaging findings. Ultrasound findings demonstrate a hyperechoic mass in the spleen, distinguishing dermoid cysts from epidermoid splenic cysts which are anechoic [7–9, 15]. Dermoid cysts can be distinguished histologically through a simple stratified epithelial lining with sebaceous portions as well as sclerotic and hyaline changes present in the connective tissue of the cyst [15]. This diverges from the characteristic appearance of splenic epidermoid cysts which demonstrate stratified squamous epithelial lining [6, 15]. However, both epidermoid and dermoid splenic cysts alike may display mucous cells along their lining [6, 16]. Dermoid cysts can also present with varied contents such as hair shafts and pulp-like material further distinguishing them from splenic epidermoid cysts [16, 17].

While the origin of epidermoid splenic cysts is still under investigation, previous case reports and research all agree that epidermoid splenic cysts are congenital, primary, nontraumatic, benign cysts [1–4]. Epidermoid cysts are postulated to derive from either mesothelial lined cysts or mesothelial cell misfolding in utero, as evidence by mesothelial cell incorporation into areas normally occupied primarily by squamous epithelial cells. With immunohistochemistry, epidermoid splenic cysts display stratified squamous epithelial cell lining with focal areas of metaplasia, indicating possible divergence from mesothelial lined cysts [6]. This incorporation of mesothelial cells is a prominent determining factor identifying splenic epidermoid cysts and is seen in their positive immunohistochemistry staining for CEA [6]. These cysts are predominately discovered in young female patients; however, neither race nor descent is a distinguishing factor. Smaller cysts generally are asymptomatic and are discovered incidentally on imaging, while larger cysts present with abdominal symptoms, such as fullness, pain, and a tender palpable mass [2, 4]. To efficiently treat epidermoid splenic cysts, one must perform partial or complete surgical removal of the spleen, as histology is crucial to confirm the diagnosis.

Previously reported cases of epidermoid splenic cysts have not been associated with congenital wandering spleen. Wandering spleen is in itself a rare random occurrence, characteristically found by imaging studies as an accidental discovery or as part of an evaluation for acute abdominal pain [18]. With wandering spleen, the spleen is hypermobile and displaces from its normal anatomic location, generally moving into the pelvic region. Due to potential life-threatening torsion of the vascular pedicle, surgery is considered the optimal treatment [1–5]. The rare finding of a congenital wandering spleen coinciding with a congenital splenic epidermoid cyst is presented. The relationship between these two seemingly disparate entities is unknown in this patient but are probably more than coincidental reflecting an incident in utero involving misplaced mesothelial cells or mesothelial progenitor cells. Thus, it might be informative to histologically evaluate extirpated incidental congenital wandering spleens to search for misplaced mesothelial cells, mesothelial lined cysts, and epidermoid cysts.
